# Supplementary material for: The Early Life Microbiota Is Not a Major Factor Underlying the Susceptibility to Postweaning Diarrhea in Piglets
Source: Microbiol Spectr. 2023 Jun 26;11(4):e00694-23. doi: 10.1128/spectrum.00694-23 (PMC10433861; doi:10.1128/spectrum.00694-23)
Supplement: Supplemental file 2 — Legends of Tables S1 to S8. Download spectrum.00694-23-s0002.pdf, PDF file, 0.1 MB [file spectrum.00694-23-s0002.pdf]

LEGENDS OF SUPPLEMENTAL TABLES

Table S1: List of samples used for microbiota analysis and corresponding metadata. Sequencing reads were deposited in the National Center for Biotechnology Center for Biotechnology Information Sequence (PRJNA591810 and PRJNA904234). Accession number of each sample is provided in column A. Sample name and metadata are presented in columns B-I. PWD: postweaning diarrhea.

Table S2: Sequences of primers used for analysis of gene expression in colon organoids by qPCR.

Table S3: α-diversity. The gut microbiota was analyzed by 16S rRNA gene amplicon sequencing at postnatal day 13 in suckling piglets that later developed or not postweaning diarrhea (PWD). Data were analyzed with a linear mixed model (fixed effects: PWD, sex and their interaction, random effects: farm and sow). Least-square means were compared within each sex. Groups labeled without a common letter differ, $P<0.05$.

Table S4: Relative abundance of bacterial phyla. The gut microbiota was analyzed by 16S rRNA gene amplicon sequencing at postnatal day 13 in suckling piglets that later developed or not postweaning diarrhea (PWD). Results are presented for the taxa which relative abundance was over 0.5% in at least one group. Data were analyzed with a linear mixed model (fixed effects: PWD, sex and their interaction, random effects: farm and sow). Least-square means were

compared within each sex for taxa with a significant interaction between PWD and sex. Groups labeled without a common letter differ, P<0.05.

Table S5: Relative abundance of bacterial families. The gut microbiota was analyzed by 16S rRNA gene amplicon sequencing at postnatal day 13 in suckling piglets that later developed or not postweaning diarrhea (PWD). Results are presented for the taxa which relative abundance was over 0.5% in at least one group. Data were analyzed with a linear mixed model (fixed effects: PWD, sex and their interaction, random effects: farm and sow). Least-square means were compared within each sex for taxa with a significant interaction between PWD and sex. Groups labeled without a common letter differ, P<0.05.

Table S6: Relative abundance of bacterial genera. The gut microbiota was analyzed by 16S rRNA gene amplicon sequencing at postnatal day 13 in suckling piglets that later developed or not postweaning diarrhea (PWD). Results are presented for the taxa which relative abundance was over 0.5% in at least one group. Data were analyzed with a linear mixed model (fixed effects: PWD, sex and their interaction, random effects: farm and sow). Least-square means were compared within each sex for taxa with a significant interaction between PWD and sex. Groups labeled without a common letter differ, P<0.05.

Table S7: Relative abundance of predicted functions of the microbiota. The gut microbiota functions were predicted with PICRUSt2 at postnatal day 13 in suckling piglets that later developed or not postweaning diarrhea (PWD). Data were analyzed with a linear mixed model (fixed effects: PWD, sex and their interaction, random effects: farm and sow). Least-square means

were compared within each sex for taxa with a significant interaction between PWD and sex. Groups labeled without a common letter differ, P<0.05.

Table S8: Relative abundance of metabolites. The fecal metabolome was analyzed by nuclear magnetic resonance-based metabolomics at postnatal day 13 in suckling piglets that later developed or not postweaning diarrhea (PWD). Data were analyzed with a linear mixed model (fixed effects: PWD, random effects: farm and sow). There was no main effect of sex or PWD x Sex interaction for metabolites. Groups labeled without a common letter differ, P<0.05.
